# Transactions of the Pa. State Medical Society

**Published:** 1872-10

**Authors:** 


*Transactions of The Medical Society of the State of Pennsylvania, at its Twenty-Third Annual Session.* Franklin, Pa., June 1872.

The annual volume of Transactions published by the Medical Society of Pennsylvania is always full of valuable suggestions and interesting papers.

The address of the President Dr. J. S. Crawford is an able and instructive paper. The speaker departs somewhat from the usual course pursued in such an address and treats his hearers to an eloquent review of the past of the Medical profession and some valuable warnings and predictions as to its future. The whole volume is an honor to the Society, and evinces their enthusiasm in matters of Professional advancement.

